# A panoramic view of the virosphere in three wastewater treatment plants by integrating viral‐like particle‐concentrated and traditional non‐concentrated metagenomic approaches

**DOI:** 10.1002/imt2.188

**Published:** 2024-03-29

**Authors:** Jiayu Zhang, Aixi Tang, Tao Jin, Deshou Sun, Fangliang Guo, Huaxin Lei, Lin Lin, Wensheng Shu, Pingfeng Yu, Xiaoyan Li, Bing Li

**Affiliations:** ^1^ Institute of Environment and Ecology, Tsinghua Shenzhen International Graduate School Tsinghua University Shenzhen China; ^2^ Research Center for Eco‐Environmental Engineering Dongguan University of Technology Dongguan China; ^3^ Guangdong Magigene Biotechnology Co., Ltd. Shenzhen China; ^4^ Shenzhen Tongchen Biotechnology Co., Limited Shenzhen China; ^5^ Institute of Ecological Science, Guangzhou Key Laboratory of Subtropical Biodiversity and Biomonitoring, Guangdong Provincial Key Laboratory of Biotechnology for Plant Development, School of Life Sciences South China Normal University Guangzhou China; ^6^ College of Environmental and Resource Sciences Zhejiang University Hangzhou China

**Keywords:** antibiotic resistance gene, auxiliary metabolic gene, metagenome, virome, virus, wastewater treatment system

## Abstract

Wastewater biotreatment systems harbor a rich diversity of microorganisms, and the effectiveness of biotreatment systems largely depends on the activity of these microorganisms. Specifically, viruses play a crucial role in altering microbial behavior and metabolic processes throughout their infection phases, an aspect that has recently attracted considerable interest. Two metagenomic approaches, viral‐like particle‐concentrated (VPC, representing free viral‐like particles) and non‐concentrated (NC, representing the cellular fraction), were employed to assess their efficacy in revealing virome characteristics, including taxonomy, diversity, host interactions, lifestyle, dynamics, and functional genes across processing units of three wastewater treatment plants (WWTPs). Our findings indicate that each approach offers unique insights into the viral community and functional composition. Their combined use proved effective in elucidating WWTP viromes. We identified nearly 50,000 viral contigs, with Cressdnaviricota and Uroviricota being the predominant phyla in the VPC and NC fractions, respectively. Notably, two pathogenic viral families, Asfarviridae and Adenoviridae, were commonly found in these WWTPs. We also observed significant differences in the viromes of WWTPs processing different types of wastewater. Additionally, various phage‐derived auxiliary metabolic genes (AMGs) were active at the RNA level, contributing to the metabolism of the microbial community, particularly in carbon, sulfur, and phosphorus cycling. Moreover, we identified 29 virus‐carried antibiotic resistance genes (ARGs) with potential for host transfer, highlighting the role of viruses in spreading ARGs in the environment. Overall, this study provides a detailed and integrated view of the virosphere in three WWTPs through the application of VPC and NC metagenomic approaches. Our findings enhance the understanding of viral communities, offering valuable insights for optimizing the operation and regulation of wastewater treatment systems.

## Highlights

- This study comprehensively compared viral‐like particle‐concentrated (VPC) and non‐concentrated (NC) metagenomic approaches in virome investigation, highlighting their respective efficacies in detecting different viral communities and functional elements in wastewater treatment plants (WWTPs).
- Eukaryotic viruses belonging to Cressdnaviricota were the most prevalent in VPC metagenomes, while bacterial viruses belonging to Uroviricota were the most abundant in NC metagenomes.
- Diverse phage‐born auxiliary metabolic genes (AMGs) were discovered in the virome of WWTPs, and a large array of AMGs involved in biogeochemical cycles like carbon, sulfur, and phosphorus cycling showed transcriptional activation in wastewater treatment systems.
- A tiny proportion (less than 0.08% of viral operational taxonomic units) of viruses was discovered to harbor antibiotic resistance genes (ARGs) (29 phage‐born ARGs) only in the cellular fraction, and phage‐born ARG types primarily included macrolide–lincosamide–streptogramin, tetracycline, and aminoglycoside resistance.

## Graphical abstract

Our comparative analysis unveiled the distinct advantages of two methods in capturing the complexity of viral communities and their functions in wastewater treatment plants. The non‐concentrated (NC) metagenomic approach identified a higher number of viral contigs, though it had a lower yield of high‐quality contigs. In contrast, the viral‐like particle‐concentrated (VPC) metagenomic method outperformed in recovering a larger proportion of high‐quality viral contigs, and significantly enriched free eukaryotic viruses. Notably, the phage‐derived auxiliary metabolic genes and antibiotic resistance genes were predominantly located in the NC metagenomes rather than the VPC metagenomes. Overall, the integration of both VPC and NC metagenomic approaches offers a robust framework for a thorough and nuanced exploration of viral communities in wastewater environments, contributing valuable insights for the advancement of wastewater treatment technologies.

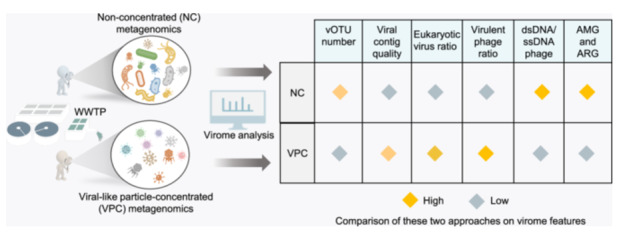

## INTRODUCTION

Municipal and agricultural wastewater, notably from livestock and poultry farming, is characterized by high chemical oxygen demand, reaching hundreds to thousands of mg/L. This eutrophic environment is a rich reservoir of diverse microbes, including protists, fungi, bacteria, archaea, and viruses [1]. The microbial communities present in wastewater, constantly replenished by inflows, are instrumental in shaping the biotic landscape of various wastewater treatment plant (WWTP) units. Interestingly, these microbial populations in raw sewage, originating from municipal or livestock sources, serve as valuable indicators for assessing the health and characteristics of human or livestock populations in catchment areas, as they reflect the fecal microbial community traits [2, 3]. Numerous studies have extensively explored the diversity and genomic attributes of bacteria and archaea in WWTPs [4, 5]. For instance, Wang et al. [4] delved into the successional dynamics of bacteria and archaea in activated sludge (AS) in a WWTP over a 9‐year period through metagenomic analysis. However, our understanding of viruses in WWTPs remains limited [1].

Viruses, the most abundant and diverse biological entities on Earth, exhibit considerable variation in morphology, size, and genomic organization. Their concentrations in wastewater systems are estimated to be around 10^8^/mL, significantly higher than in other aquatic environments [2, 6]. A majority of viruses belong to bacterial or archaeal phages (also called bacteriophages). Phages notably outnumber their prokaryotic hosts, directly impacting prokaryotic communities through infection followed by lysis or lysogeny [2, 6]. The release of cellular contents by lytic infection of phages will occur in 20%–40% of bacteria per day in aquatic environments, which has a noticeable impact on organic carbon and other nutrient cycles in Earth's biosphere [7]. Moreover, phages can reprogram the metabolic pathways of their hosts by transferring auxiliary metabolic genes (AMGs), thereby impacting the efficiency of biotreatment systems in wastewater [1, 8]. Horizontal gene transfer, facilitated by viral transduction, is crucial in sharing genetic material among bacterial taxa and notable in the spread of antibiotic resistance genes (ARGs), posing challenges to antimicrobial treatment strategies [9]. Notably, eukaryotic viruses, potentially originating from humans or other eukaryotes, have also been detected in sewage, indicating their role in forecasting viral epidemics such as swine flu and COVID‐19 [10].

Advancements in high‐throughput sequencing technologies have revolutionized the study of microbial communities by enabling comprehensive metagenomic sequencing at the DNA level [11]. When investigating viromes via metagenomics, researchers typically employ either viral‐like particle‐concentrated (VPC, targeting free viral‐like particle fraction) or non‐concentrated (NC, targeting cellular fraction) sample preparation methods [12]. The NC approach, which includes techniques like centrifugation or filtration, is relatively straightforward but tends to capture a broader array of larger cells, such as fungi and bacteria, alongside a smaller viral fraction (1%–19%) [13, 14]. Due to its operational simplicity and ability to provide a holistic view of microbial communities, NC metagenomics has been extensively used in various studies for environmental viromes [15, 16]. In contrast, methods like adsorption‐elution and ultracentrifugation, designed to concentrate free‐living viruses, could potentially enhance effectiveness in virome analysis. These techniques are effective in removing prokaryotic and eukaryotic cells, thereby enriching viral content and yielding more accurate virome data [1, 12]. For instance, the chemical flocculation method using FeCl_3_, developed by John et al. [17] has gained widespread acceptance for concentrating viruses [1]. These contrasting approaches underscore the evolving landscape of virome research, highlighting the importance of selecting appropriate methodologies based on specific study goals and environmental contexts.

Despite these advancements, gaps remain in our understanding of the virome acquired through different sample preparation methods. This study aims to address these gaps by collecting microbial samples from three full‐scale WWTPs, each processing different wastewater types (duckery, swine, and municipal). We aimed to (1) compare the VPC and NC methods in virome exploration, (2) characterize the panoramic view of the viral communities in these diverse WWTPs, and (3) uncover the active roles of viruses in wastewater treatment systems using meta‐transcriptomics.

## RESULTS AND DISCUSSION

### Comparative analysis of viral communities in wastewater treatment processes: NC versus VPC metagenomic approaches

**Figure 1:**
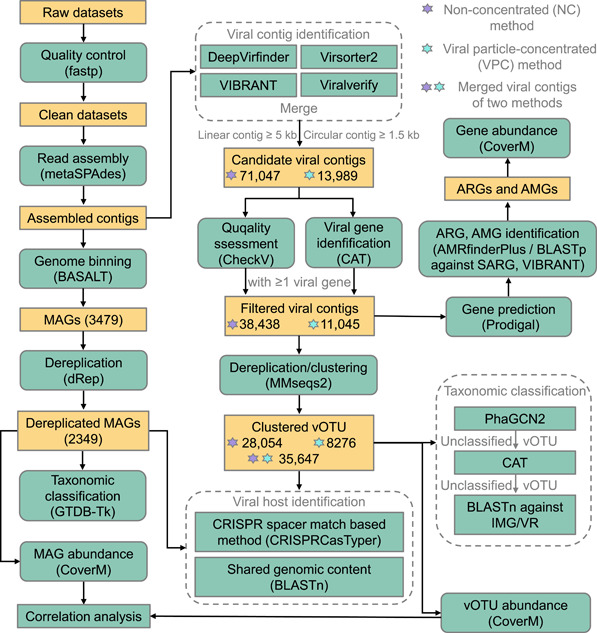
Metagenomic analysis pipeline for metagenome‐assembled genome (MAG) recovery, viral contig identification, and taxonomic classification. The primary analysis tools and the count of viral contigs or MAGs are delineated on the diagram. AMG, auxiliary metabolic gene; ARG, antibiotic resistance gene; NC, non‐concentrated; vOTU, viral operational taxonomic unit; VPC, viral‐like particle‐concentrated.

**Figure 2:**
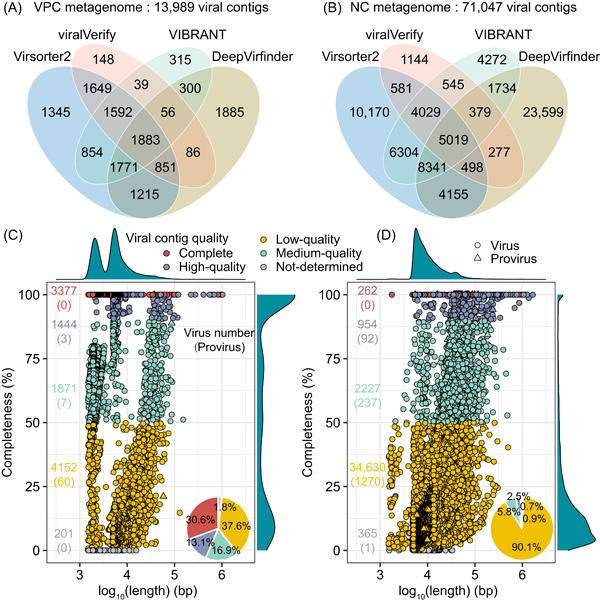
Quantity and quality of viral contigs derived from viral‐like particle‐concentrated (VPC) and non‐concentrated (NC) metagenomes. (A) and (B) Intersection of candidate viral contigs identified by four virus detection tools. (C) and (D) Contig quality of viruses identified from VPC (C) and NC (D) metagenomes. The number of viruses including proviruses (noted in brackets) at each quality level is annotated. Kernel density plots provide data distribution. Pie charts depict the proportion of viruses at each quality level.

In this study, a comprehensive analysis of viral communities across three full‐scale WWTPs was conducted using both VPC and NC metagenomic approaches (Figure 1). These WWTPs treated wastewater from duck farms (WWTP A), swine farms (WWTP B), and municipal sources (WWTP C). We identified a total of 13,989 and 71,047 viral contig candidates from assemblies of VPC and NC metagenomes, respectively (Figure 2A,B). Remarkably, 92.4% of these viral contigs were identified by Virsorter2 and DeepVirfinder, indicating the effectiveness of these tools in detecting viral contigs (Figure 2A,B). Through filtration by CheckV and CAT, there were 11,045 and 38,438 viral contigs for VPC and NC metagenomes, respectively (Figure 1). The NC metagenomes yielded longer viral contigs, with average lengths and N50 sizes of 16,250 bp and 24,310 bp, compared to 8149 bp and 11,929 bp for VPC metagenomes. A notable finding was the detection of 70 and 1600 proviruses by VPC and NC methods, respectively (Figure 2C,D), highlighting the NC method's superiority in identifying proviruses integrating into host cell DNA. The NC method's focus on cell‐bound viruses likely contributes to this proficiency. Interestingly, despite fewer viral contigs identified in VPC metagenomes, the proportion of complete (30.6%) and high‐quality (13.1%) viral contigs was significantly higher than in NC metagenomes (Figure 2C,D). The percentage of VPC metagenomic reads aligned with viral contigs varied between 22.1% and 97.4%, whereas the alignment percentage for NC metagenomic reads was considerably lower, ranging from 0.9% to 17.4% (Figure S1). Virus concentration via size fractionation approach (i.e., VPC approach) before DNA extraction could decrease the component of nonviral DNA to generate more viral reads in metagenomes, thus enabling the recovery of viral contigs with high quality [17].

About 56.9% and 40.6% of viral contigs in VPC and NC viromes could be classified by our pipeline (Figure S2A). Over one‐third of these classified contigs were traced to bacterial viruses. Notably, a higher proportion of eukaryotic viruses was detected in VPC metagenomes (19.4%) compared to NC metagenomes (4.3%) (Figure S2B). The distribution of viral contigs at the phylum level varied between the two methods, with VPC metagenomes predominantly showing Phixviricota, Cressdnaviricota, and Uroviricota, and NC metagenomes being largely comprised of Uroviricota (Figure S2C). An intersection analysis at the class and family levels revealed 27 viral classes and 97 families, with some unique ones to each method (Figure S2D). These results demonstrate that the VPC and NC metagenomic approaches are complementary for virome characterization in WWTPs. In contrast, Santos‐Medellin et al. [13] found that VPC metagenomes were more effective than NC metagenomes in uncovering viral communities in agricultural soil, suggesting a broader richness and diversity of viruses. Whereas, our data manifested that VPC metagenomes excelled at high‐quality virus detection, while NC metagenomes were superior for provirus detection. Moreover, each method recovered unique viral taxonomies, suggesting that an integrated approach combining VPC and NC metagenomics would offer a more robust and comprehensive strategy for characterizing the virosphere of WWTPs.

### Dynamics of viruses infecting diverse hosts across wastewater treatment processes: NC versus VPC metagenomic approaches

**Figure 3:**
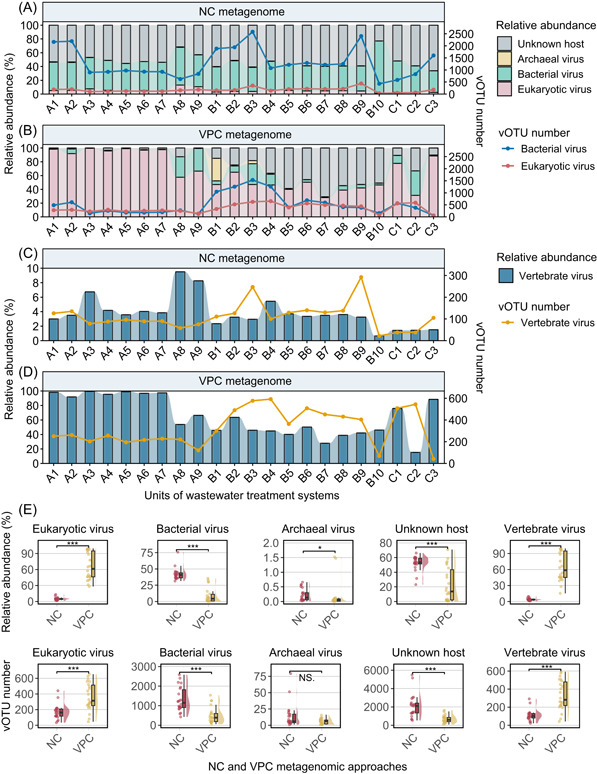
Dynamics of viruses infecting different types of hosts across the wastewater treatment streams. (A) and (B) Relative abundance and diversity of viruses infecting archaea, bacteria, eukaryotes, and unknown hosts in NC and VPC metagenomes. The lines indicate vOTU numbers. (C) and (D) Relative abundance and diversity of vertebrate viruses. The lines indicate vOTU numbers. (E) Comparison of viral abundance and diversity revealed by NC and VPC metagenomic approaches. ﻿﻿﻿﻿﻿﻿* ﻿﻿indicates *p* < 0.01. ** indicates *p* < 0.005. *** indicates *p* < 0.001﻿.﻿ NS. indicates no significant difference. NC, non‐concentrated; vOTU, viral operational taxonomic unit; VPC, viral‐like particle‐concentrated.

In this study, we investigated the dynamics of viruses infecting different host types across wastewater treatment processes using VPC and NC metagenomic approaches. Viral contigs from three full‐scale WWTPs were clustered into viral operational taxonomic units (vOTUs), amounting to 8276 and 28,054 vOTUs for VPC and NC metagenomes, respectively (Figure 1). The combined analysis of VPC and NC metagenomes yielded a total of 35,647 vOTUs. Our findings indicated a clear dominance of bacteriophages in NC metagenomes and eukaryotic viruses, particularly those infecting vertebrates, in VPC metagenomes (Figure 3A,B,E). The NC method, focusing on the bacterial cellular fraction, was found to be more effective for studying bacteriophages in wastewater. Conversely, the VPC method, targeting the free viral particle fraction, showed a higher efficacy in investigating free eukaryotic viruses, especially those infecting vertebrates from animals or humans.

For WWTPs processing duckery and swine wastewater (WWTPs A and B), a decrease in the number of vOTUs of bacterial viruses was observed through the treatment processes, yet their relative abundance did not follow a predictable pattern (Figure 3A). The relative abundance of free‐living eukaryotic viruses, including those infecting vertebrates, was significantly higher in WWTP A compared to WWTPs B and C. However, their diversity displayed an inverse trend (*p* < 0.001) (Figure 3B,D). Wastewater environments are generally inhospitable for eukaryotic viruses, given the lack or scarcity of suitable hosts, especially for vertebrate viruses [18]. We observed a significant reduction in the diversity of eukaryotic viruses, particularly those infecting vertebrates, in the effluents of all three WWTPs (*p* < 0.001) (Figure 3B,D), suggesting some efficacy of wastewater treatment systems in removing these viruses. Notably, the wastewater treatment systems did not completely remove vertebrate viruses, and their relative abundance remained high in the effluents, pointing to ongoing health risks (Figure 3C,D). Several studies have indicated that the wastewater treatment system showed limited efficacy in vertebrate virus removal as they could be detected both in the effluent and receiving water [19, 20].

The viral structure is a crucial determinant for their survival across wastewater treatment processes. For instance, double‐stranded and nonenveloped viruses, such as enteric viruses, generally exhibit higher resistance to UV radiation disinfection compared to single‐stranded or enveloped viruses [18]. Some treatment processes can inadvertently decrease virus inactivation. For example, virus adsorption to solids aids removal but also provides protection from inactivation [18]. Furthermore, the persistence of viruses in wastewater contributes to their waterborne transmission. In this study, the predominant viruses detected by the integration of NC and VPC metagenomic approaches were double‐stranded DNA (dsDNA, 32.5%) and single‐stranded DNA (ssDNA, 11.2%) viruses (Figure S3A). The cellular fraction was mainly comprised of dsDNA viruses, whereas the free viral particle fraction was dominated by ssDNA viruses (Figure S3B). Notably, the abundance of ssDNA viruses significantly declined in the effluent of WWTP A (Figure S3B), indicating the effective inactivation of ssDNA viruses by the disinfection process. In contrast, WWTP C's treatment system showed less efficacy in inactivating ssDNA viruses. These observations highlighted the ongoing challenge of achieving efficient viral inactivation in wastewater treatment systems.

### Efficacy in detecting viruses of extreme sizes in wastewater treatment processes: NC versus VPC metagenomic approaches

This study further evaluated the ability of VPC and NC metagenomic approaches to detect viruses at extreme sizes (Figure S4). Focusing on small viruses, such as those in the Circoviridae and Parvoviridae families, typically under 30 nm in diameter, we found that the VPC approach was more effective in detecting Circoviridae populations than the NC approach (*p* < 0.001) (Figure S4B). This is consistent with the fact that many Circoviridae, small vertebrate‐infecting viruses, are frequently detected in wastewater [21, 22]. For Parvoviridae, however, there was no significant difference regarding the number of vOTUs detected between the two methods (*p* > 0.05) (Figure S4B). Regarding larger viruses, such as those in the Mimiviridae family (giant viruses), known for their diverse large dsDNA viruses with sizes ranging from 140 to 750 nm [23], we observed a different pattern. The NC metagenomic approach identified more vOTUs classified into Mimiviridae compared to the VPC approach (Figure S4B). This might be attributed to the larger size of some giant viruses (>220 nm), which were potentially excluded from the free viral particle fraction during the VPC process. Therefore, for virome research, it is crucial to select appropriate processing methods based on the size of the viral populations or to refine viral particle enrichment methods to ensure a comprehensive analysis.

### Dynamics of virulent and temperate phages across wastewater treatment processes: NC versus VPC metagenomic approaches

Our analysis revealed distinct differences in the proportions of virulent and temperate phages within cellular (NC metagenomes) and free viral particle (VPC metagenomes) fractions. Specifically, virulent phages constituted about 56.1% of phages in cellular fraction, while their proportion in free viral particle fraction averaged 87.7% (Figure S5A,B). The diversity and relative abundance of virulent phages were significantly higher in the free viral particle fraction compared to the cellular fraction (*p* < 0.001) (Figure S5C,D). Virulent phages exclusively follow the lytic cycle, wherein they replicate and cause lysis of the host cells [24]. Their replication through the lytic cycle, culminating in their release into the extracellular environment, accounts for their predominance in the free viral particle fraction. Understanding the lifestyle of phages is increasingly important for environmental engineers, particularly in the context of wastewater bioprocesses. Despite their known presence and abundance, the ecological role, potential benefits, and impacts of phages on wastewater biological processes remain not fully comprehended [25]. Our findings underscore the importance of method selection in metagenomic studies focusing on phage lifestyles. The choice between NC and VPC metagenomic approaches can significantly influence the observed outcomes, highlighting the need for careful consideration in method selection for specific research objectives.

### Viral diversity across wastewater treatment streams: NC versus VPC metagenomic approaches

**Figure 4:**
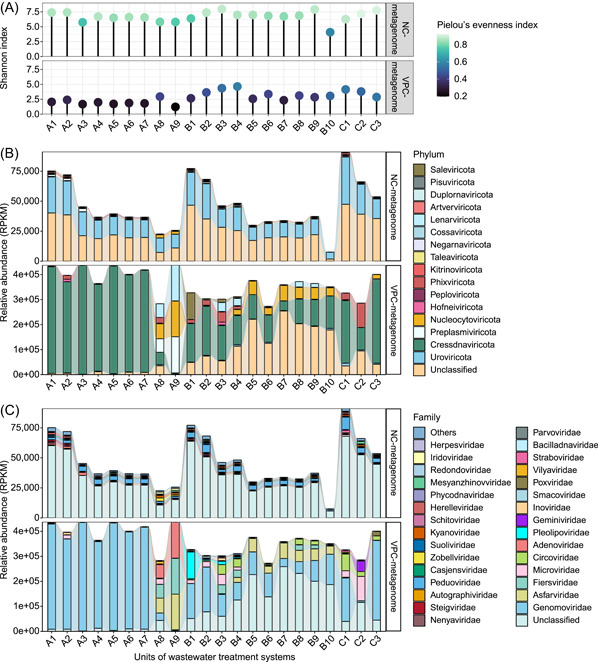
Diversity and relative abundance of viruses in each sample. (A) Alpha diversity of viruses. (B) Viral composition at the phylum level. (C) Viral composition at the family level. A1–A9 represent samples collected from WWTP A treating duckery wastewater. B1–B10 represent samples collected from WWTP B treating swine wastewater. C1–C3 represent samples collected from WWTP C treating municipal wastewater. WWTP, wastewater treatment plant.

In examining the diversity of viral communities across wastewater treatment streams, our study revealed notable differences between VPC and NC metagenomic approaches. The NC metagenomes displayed a significantly richer viral diversity compared to the VPC metagenomes. This was evident in the alpha diversity indices, including the Shannon and Pielou's evenness indices, which were markedly higher in NC metagenomes (*p* < 0.001) (Figure 4A). In terms of beta diversity, there was also a significant dissimilarity between the viral communities characterized by the VPC and NC methods (*p* < 0.001) (Figure S6A). This divergence is attributable to the distinct focus of each method: VPC primarily sequences the free viral‐like particle fraction, while NC targets the cellular fraction. Further analysis of samples from WWTPs A and B using PCoA revealed significant separations (*p* < 0.01), indicating that the type of wastewater significantly influenced the formation of virosphere in treatment systems (Figure S6A). Additionally, a remarkable difference in the NC virome at the family level was observed among influent, sludge, and effluent samples (*p* < 0.05) (Figure S6B). This finding aligns with Li et al. [26], who reported significant variations in viral compositions across influent, sludge, and effluent. Activated sludge in WWTPs, known for its high microbial density, forming biomass in the range of 2–50 g/L in granules or condensed flocs, potentially influences the viral profile of wastewater effluent during the treatment process [1, 26].

### Abundant viral populations in WWTPs

We characterized the prevalent viral populations, revealing distinct differences in the abundance and variety of viruses in WWTPs. Cressdnaviricota was the most predominant phylum with an average relative abundance of 56.9 ± 33.7% in VPC metagenomes, while Uroviricota was more prevalent in NC metagenomes, averaging 40.8 ± 9.1% (Figure 4B). Cressdnaviricota comprising circular, replication proteins (Rep)‐encoding ssDNA (CRESS‐DNA) viruses consists of two classes (Arfiviricetes and Repensiviricetes) and 11 families to date [27]. In our study, 1989 viral contigs from this phylum were identified across 9 families in these three WWTPs (Figure S2C). Cressdnaviricota viruses are known for their diversity and presence in various habitats, including those associated with plants, animals, and humans [27, 28, 29].

Among the Cressdnaviricota, Genomoviridae was especially predominant in the VPC metagenomes of the three WWTPs, representing a relative abundance of 50.6 ± 36.9% (Figure 4C). In WWTP A, treating duckery sewage, Genomoviridae viruses were abundantly present, especially from the influent to the secondary aerobic tank with an average relative proportion of 96.5 ± 2.8%. Members of Genomoviridae have been reported to be associated with a wide host range, including fungi, insects, birds, mammals, plants, sewage, and sediments [27]. Members of Circoviridae were also notably abundant in the VPC samples of WWTPs processing swine and municipal sewage with an average relative proportion of 5.5%, particularly in the municipal sewage sample (Sample C1) reaching 20.4%. The significance of Circoviridae lies in its members being the smallest known eukaryotic cell‐infecting viruses, often linked with various clinical diseases in animals and a high prevalence in human populations [21, 30]. Some Circoviridae members, predominantly from birds and pigs, exhibit pathogenicity, causing developmental disorders and immune system damage linked to clinical diseases, such as infectious chicken anemia and postweaning multisystemic wasting syndrome in pigs [21]. In addition, we observed that human‐associated CRESS‐DNA viruses belonging to Redondoviridae were also abundant in the sewage of these three WWTPs with relative abundance >0.1% in VPC metagenomes. Viruses of Redondoviridae mainly colonize the human oro‐respiratory tract and have been considered to be associated with periodontitis [31].

The VPC metagenomics further revealed the presence of pathogenic viral families like Asfarviridae and Adenoviridae. Asfarviridae, containing the African swine fever virus, was detected in all samples from swine wastewater treatment systems with an average relative abundance of 8.3%. The African swine fever virus can trigger a highly contagious viral disease in pigs, with mortality rates approaching 100%, thereby significantly impacting the pig breeding industry [32]. Adenoviridae, known for infecting an extensive range of vertebrates including humans, was notably abundant in duck wastewater treatment systems, particularly in effluent samples. Adenoviridae has been discovered to be prevalent in wastewater and human‐associated viromes, and some members can cause damage to many organs like the ocular surface, throat, and lungs in vertebrates [2, 33, 34]. The Fiersviridae and Microviridae families, commonly found in gut and marine samples [35, 36], were also prevalent in various VPC samples from three WWTPs. This observation enhances our comprehension of their ecological habitats.

In contrast, NC metagenomes were dominated by dsDNA‐tailed phages of the Uroviricota phylum, particularly the Caudoviricetes class, known for their diverse, abundant, and widespread presence [37]. Uroviricota members, including Peduoviridae, Herelleviridae, Kyanoviridae, Mesyanzhinovviridae, Casjensviridae, Straboviridae, and Suoliviridae, were prevalent in NC samples from three WWTPs (Figure 4C). Peduoviridae, with an average relative abundance of 8.7 ± 3.5%, was particularly predominant in all WWTP samples. These tailed phages may play a crucial role in modulating biogeochemical cycles and bacterial metabolism through their lytic life cycle and activating functional AMGs that significantly impact sewage treatment systems [37, 38].

### Virus–prokaryote associations in WWTPs

**Figure 5:**
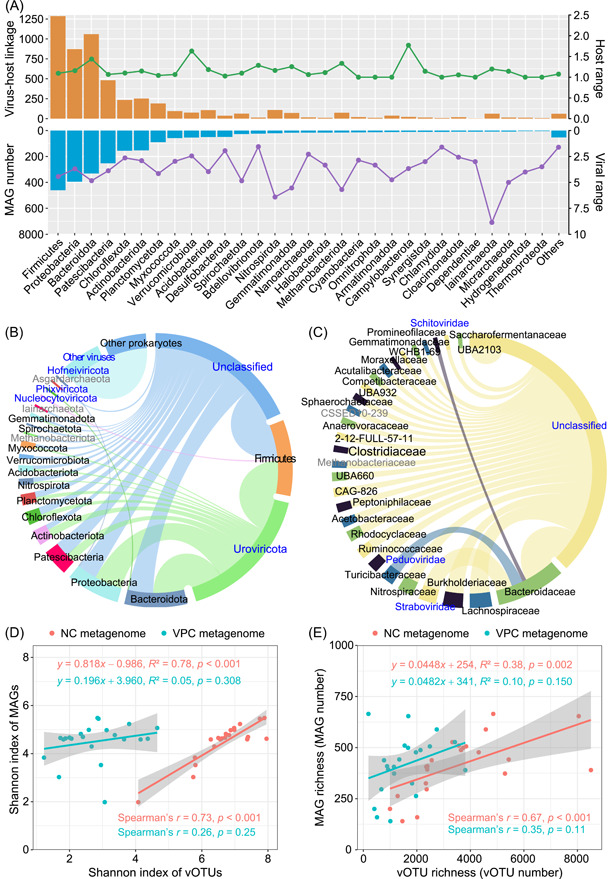
Virus–prokaryote associations in WWTPs. (A) MAG numbers and virus–host events at the phylum level. The orange column indicates the number of virus–host linkages, while the blue column indicates the number of MAGs per phylum. The purple line indicates the average viral range of MAGs per prokaryotic phylum. The green line indicates the average host range of viruses infecting each prokaryotic phylum. (B) Virus–prokaryote association at the phylum level. (C) The top 30 virus–prokaryote associations at the family level. The chord width and bar length represent the number of virus–host linkages. (D) Linear and Spearman's correlation of Shannon index of vOTUs and MAGs. (E) Linear and Spearman's correlation of richness of vOTUs and MAGs. The gray shade indicates the confidence interval of the linear correlation. MAGs, metagenome‐assembled genomes; vOTUs, viral operational taxonomic units; WWTPs, wastewater treatment plants.

In the current study, we recovered 2349 metagenome‐assembled genomes (MAGs), comprising 2277 bacteria and 72 archaea. CRISPR spacer match and shared genomic contents of prokaryotic hosts can provide evidence of past phage infection events [8]. Herein, a total of 5341 potential virus–host associations were characterized between MAGs and viral contigs according to CRISPR spacer match and shared genomic contents. Firmicutes (461 MAGs), Proteobacteria (395 MAGs), and Bacteroidota (331 MAGs) were the three most diverse phyla, and they were associated with the largest number of viral infection events in WWTPs (Figure 5A). The average host range of viruses was approximately 1.1, implicating that most phages tend to infect specific species of bacteria or archaea. This specificity can be attributed to phages recognizing and attaching to particular receptors on host cell surfaces [39]. Conversely, the viral range for these MAGs was approximately 3.9, suggesting that prokaryotes were often targeted by multiple phages. This multiplicity of infections can drive microbial evolution and adaptation [40]. In particular, Uroviricota phages were predicted to infect a wide range of hosts, notably Firmicutes, Bacteroidota, and Proteobacteria (Figure 5B). Common viral families of Uroviricota, such as Straboviridae, Peduoviridae, and Schitoviridae, were frequently linked to host infections in WWTPs (Figure 5C).

Herein, we also explored the diversity correlation between viral contigs and MAGs, and found a significant linear correlation (*p* < 0.01) between them in NC metagenomes, but not in VPC metagenomes of three WWTPs (Figure 5D,E). This suggests a closer association between viral communities and prokaryotes in the cellular fraction compared to the free viral‐like particle fraction. The concentration process might be an important interference factor for the correlation between the cell‐free viral community and the bacterial community. In recent years, the interaction between phages and prokaryotes has garnered substantial attention from environmental engineers, especially in the realm of wastewater bioprocesses, where it plays a crucial role in influencing outcomes [25].

### Functional activation of AMGs in viruses revealed by meta‐transcriptomics

**Figure 6:**
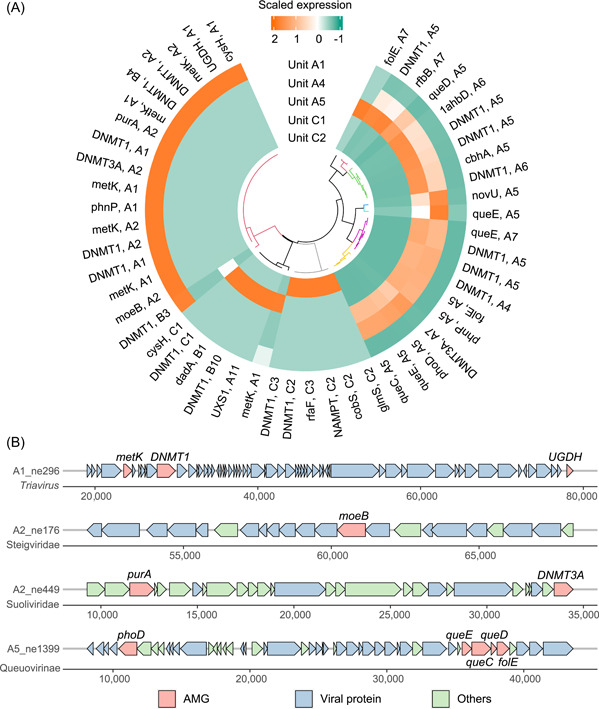
Representative auxiliary metabolic genes (AMGs) carried by viruses. (A) Expression of representative AMGs in viruses. Values are presented as scaled means of transcripts per million (TPM) values (*n* = 3). The cladogram represents clustering based on scaled TPM values of AMGs. (B) Arrangement of representative AMGs in viruses.

Meta‐transcriptomic sequencing of sewage and sludge samples from WWTPs A and C revealed the transcriptional activity of viruses, indicating their potential impact on the ecosystem within the treatment systems (Figure S7). Phage viruses harbor diverse AMGs which are extensively involved in the microbial‐driven biogeochemical cycle and even biological processes like antibiotic resistance. Phages harbor a range of AMGs involved in biogeochemical cycles and biological processes. AMGs can modulate host metabolism during infection, aiding adaptation to ecosystem fluctuations [41]. Herein, we discovered 827 and 99 putative AMGs in NC and VPC metagenomes of WWTPs, respectively (Figure S8A). NC metagenomes exhibited a wider array of AMG‐involved metabolic pathways than VPC metagenomes (Figure S8B), indicating the NC approach's effectiveness in unearthing well‐rounded AMG contents in WWTPs. These discovered AMGs were extensively involved in the metabolism of carbohydrates, amino acids, cofactors, vitamins, and so on (Figure S8B). Uroviricota phages were the major AMG carriers that harbored 64.0% of these discovered AMGs, facilitating viral fitness enhancement or host metabolism compensation. A large array of AMGs, such as *cysH*, *phoD*, *dadA*, *queC*, *queD*, *queE*, *rfbB*, *cbhA*, *UGDH*, *cobS*, *metK*, *moeB*, *glmS*, *UXS1*, and *DNMT1*, showed transcriptional activation in wastewater treatment systems (Figure 6A), implicating they were affecting host metabolisms and microbial‐driven biogeochemical cycles. AMGs related to carbohydrate metabolism, such as *UXS1* (UDP‐glucuronate decarboxylase), *glmS* (glutamine‐fructose‐6‐phosphate transaminase), *cbhA* (cellulose 1,4‐beta‐cellobiosidase), and *UGDH* (UDP glucose 6‐dehydrogenase), and those involved in sulfur and phosphorus cycling, like *cysH* (phosphoadenosine phosphosulfate reductase), *moeB* (molybdopterin‐synthase adenylyltransferase), and *phoD* (alkaline phosphatase), were activated in wastewater treatment systems. Gene *cysH* encoding phosphoadenosine phosphosulfate reductase is responsible for the reduction of 3′‐phosphoadenosine‐5′‐phosphosulfate into free sulfite, an important process in the reductive assimilation of sulfate [42]. Gene *moeB* involved in the sulfur relay system was actively expressed in a representative viral contig (contig ID: A2_ne176) of Steigviridae (Figure 6B). A variety of the discovered AMGs, such as *cobA* (cob(I)alamin adenosyltransferase), *cobT* (cobaltochelatase CobT), *cobS* (cobaltochelatase), *queC* (7‐cyano‐7‐deazaguanine synthase), *queD* (6‐pyruvoyltetrahydropterin/6‐carboxytetrahydropterin synthase), *queE* (7‐carboxy‐7‐deazaguanine synthase), and *queF* (7‐cyano‐7‐deazaguanine reductase), are involved in the biosynthesis of cofactors and vitamins, which have distinct biochemical roles in a variety of physiological processes. For instance, *cobA*, *cobS*, and *cobS* are crucial genes for cobalamin (vitamin B12) biosynthesis, and *cobS* was observed to be actively expressed by viruses in activated sludge (Unit C2). Genes *queC*, *queD*, *queE*, and *queF* are crucial components for folate (vitamin B9) biosynthesis, and *queC*, *queD*, and *queE* co‐existed in a representative phage contig (contig ID: A5_ne1399) of Queuovirinae (Figure 6B). Since numerous bacteria or archaea do not possess all gene components for a complete vitamin biosynthetic pathway [43], the expression of these viral AMGs could compensate for the deficiencies of their hosts. The expression of these viral AMGs suggests an active role of viruses in shaping microbial metabolism and influencing wastewater treatment processes.

### ARG content in the virome of wastewater treatment systems

**Figure 7:**
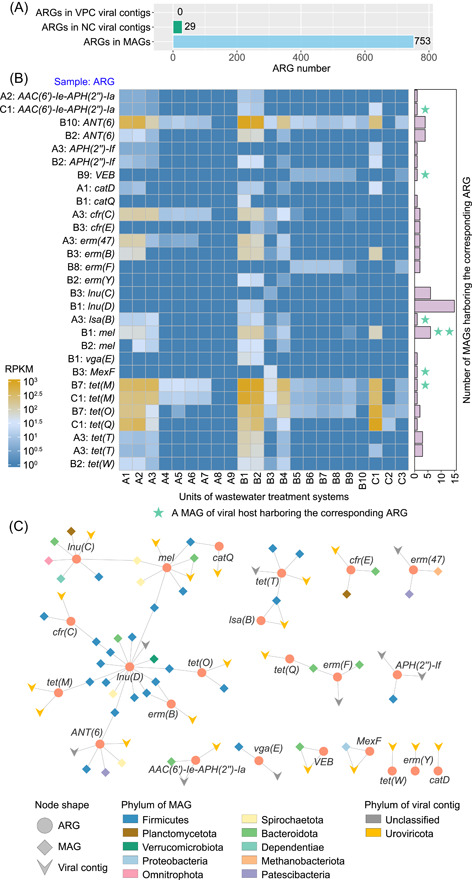
Antibiotic resistance genes (ARGs) harbored by viral contigs in NC metagenomes. (A) Numbers of ARGs harbored by NC viruses, VPC viruses, and MAGs. (B) Relative abundance of ARGs harbored by viruses. Relative abundance is presented as reads per kilobase per million mapped reads (RPKM) values. The green star symbol in the right histogram indicates an MAG of the host carrying a corresponding virus‐born ARG. (C) A network of ARGs co‐occurrence in viruses and prokaryotes. MAGs, metagenome‐assembled genomes; NC, non‐concentrated; vOTU, viral operational taxonomic unit; VPC, viral‐like particle‐concentrated.

Wastewater treatment systems are increasingly recognized as reservoirs of ARGs. ARGs are predominantly found within a wide range of bacterial genomes or plasmids and can be disseminated through horizontal gene transfer, posing significant public health challenges [44, 45]. Herein, 753 ARGs were discovered in MAGs from the metagenomes of these three WWTPs. Only 29 phage‐born ARGs were discovered by the NC metagenomic approach (Figure 7A). In contrast, no phage‐associated ARG was found by the VPC metagenomic approach. Horizontal gene transfer, facilitated by mobile genetic elements, is the primary mechanism for the transfer of ARGs across diverse taxonomic levels. Phages, as another category of mobile genetic elements, play a role in exchanging genetic material among different bacterial taxa, thereby being implicated in the transfer of ARGs [46]. Until now, there is still a controversy about whether viruses contribute significantly to the spread of ARGs. While some studies suggest viruses are key ARG reservoirs in various environments [47, 48, 49], others argue ARGs are rarely encoded by phages [50, 51]. Our findings align with the latter, showing that a small fraction (less than 0.08% of vOTUs) of viruses carried ARGs. Consistently, several reports concluded that less than 0.1% of viral populations encoded ARGs in environmental samples, such as soil, rumen, and feces [9, 50, 52]. However, the potential for virus‐mediated ARG dissemination warrants vigilance. Future studies should further investigate the role of phage viruses in horizontal ARG transfer across bacterial taxa.

In this study, 29 phage‐born ARGs included macrolide–lincosamide–streptogramin (12 ARGs), tetracycline (seven ARGs), aminoglycoside (six ARGs), chloramphenicol (two ARGs), beta‐lactam (one ARG), and multidrug resistance (one ARG) types (Figure 7B). The arrangement of representative ARGs in viral contigs is depicted in Figure S9. Among these phage‐carried ARGs, *ANT*(6), *cfr*(C), *erm*(47), *erm*(B), *mel*, *tet*(M), *tet*(O), *tet*(Q), and *tet*(T) were abundant in sewage samples. The pattern of ARG sharing between viral contigs and MAGs revealed that the lincosamide nucleotidyltransferase‐encoding gene *lnu*(D) exhibited the most extensive linkages between bacteria and viruses (Figure 7C). Macrolide–lincosamide–streptogramin, tetracycline, and aminoglycoside resistance genes were the primary ARG types carried by viruses in WWTPs, aligning with reports of their presence in diverse environmental viromes in soil, sediment, and rumen [9, 52, 53]. These ARG types are prevalent in livestock and municipal wastewater [54, 55], and their spread is mediated by diverse mobile genetic elements including gene transduction by phages [56]. Most of the 29 ARG‐carrying viruses were classified into the phylum Uroviricota (Figure 7C). Noteworthily, 25 of these phage‐carried ARGs were detected in bacterial MAGs, including seven hosts of these ARG‐carrying phages. The co‐occurrence of ARGs was mainly identified in the bacterial phylum Firmicutes and the viral phylum Uroviricota. Considering that Uroviricota phages exhibit high abundance and diversity in WWTPs (Figures 4B and S2C) and are prevalent across various environments, their potential carriage of ARGs may contribute to the dissemination of ARGs in wastewater treatment systems, thereby elevating the risks associated with antibiotic resistance [47, 57]. Collectively, Uroviricota phages emerged as the primary carriers of both AMGs and ARGs in WWTPs. The prevalence of Uroviricota in the cellular fraction underscores why NC metagenomics outperformed VPC metagenomics in uncovering the contents of AMGs and ARGs.

## CONCLUSIONS

This study conducted a comparative analysis of viral‐like VPC and NC metagenomic approaches to elucidate the viromes in WWTPs. Our findings highlight the distinct advantages of each method in understanding viral communities and their functional content in WWTP environments. The NC metagenomic approach revealed a larger number of viral contigs (38,438), including a higher count of proviruses (1600), but with a smaller proportion (3.2%) of high‐quality viral contigs. In contrast, the VPC approach excelled at recovering high‐quality viral contigs (43.6%). The viral communities identified through the VPC method, focusing on free viral‐like particle fractions, differed significantly from those obtained via the NC method, which targets the cellular fraction. In VPC viromes, eukaryotic viruses, particularly from the phylum Cressdnaviricota, were predominant, whereas Uroviricota phages were the dominant group in NC viromes. Notably, VPC metagenomes revealed the prevalence of pathogenic viral families, such as Asfarviridae and Adenoviridae, within wastewater treatment systems. A significant aspect of our study is the identification of a large number of unclassified viral contigs in WWTP viromes, underscoring the presence of vast, yet‐to‐be‐explored viral diversity or “viral dark matter” in these systems. The NC approach demonstrated superiority in exploring the functionality of the WWTP virome, providing more comprehensive insights into AMGs and ARGs compared to the VPC approach. Collectively, employing both VPC and NC metagenomic methods concurrently offers a robust strategy for a more complete understanding of virosphere in wastewater treatment systems. With these methods employed, this study provides new insights into the crucial yet often overlooked role of viruses in WWTPs, emphasizing their significance in the development of innovative wastewater treatment technologies.

## METHODS

### Sample collection and viral‐like particle concentration

To compare viral communities in WWTPs processing various types of wastewater, we collected 23 wastewater or sludge samples, each roughly 5 L, from every processing unit of three full‐scale WWTPs. These WWTPs treat wastewater from duck farms (WWTP A), swine farms (WWTP B), and municipal sources (WWTP C) located in Yunfu and Shenzhen, Guangdong Province, China. The specific details of these samples are presented in Table S1. For traditional NC metagenomic sequencing, approximately 20 mL of each sample was subjected to centrifugation at 10,000 g for 5 min, except for effluent samples. The microbial cells from about 200 mL of effluent were collected on 0.22 μm Durapore membrane filters (Millipore) using an aspirator filter pump. The free virus DNA fraction was processed using a modified viral‐like particle‐concentrated (VPC) method based on John et al. [17]. Briefly, the filtrate through the 0.22 µm filters was mixed with AlCl_3_ (final concentration of 20 mg/L Al^3+^) to precipitate viral‐like particles. The precipitate containing viral‐like particles was collected on a 0.22 µm filter and then resuspended in the ascorbate buffer (pH = 6.0). DNase I and RNase A at final concentrations of 10 and 1 U/mL were added to digest exogenous nucleic acid. The enzyme reaction was terminated by ethylene diamine tetraacetic acid (EDTA) and ethylene glycol tetraacetic acid (EGTA) at final concentrations of 100 mM. Finally, the supernatant was collected by centrifugation at 2000 g for 5 min and stored at −20°C for subsequent viral DNA extraction.

### Nucleic acid extraction and sequencing

The cellular DNA of samples was extracted using the FastDNA^TM^ Spin Kit for Soil (MP Biomedicals) adhering to the manufacturer's protocol. The viral DNA of VPC samples was extracted using the MiniBEST Viral DNA Extraction Kit (TaKaRa). To increase the viral DNA yield from VPC samples, the whole genome was amplified with illustra^TM^ Ready‐To‐Go^TM^ GenomiPhi^TM^ V3 DNA Amplification Kit (GE Healthcare). The metagenomic sequencing libraries were constructed using NEB Next® Ultra™ DNA Library Prep Kit for Illumina® (New England Biolabs) following the manufacturer's guidelines. Finally, 150 bp paired‐end reads were generated through sequencing on an Illumina Novaseq. 6000 platform at Magigene. Sequencing was performed on the Illumina NovaSeq. 6000 platform at Magigene, producing 150 bp paired‐end reads. The sequencing depths achieved approximately 40 Gb for cellular DNA and 10 Gb for viral DNA.

The RNA of wastewater or sludge samples in WWTPs A and C was extracted using the Soil RNA Mini Kit (OMEGA Bio‐tek). The strand‐specific meta‐transcriptomic library was prepared using NEBNext®UltraTM Directional RNA Library Prep Kit for Illumina (NEB). Meta‐transcriptomic sequencing was conducted using a 150 bp paired‐end strategy, achieving a sequencing depth of approximately 20 Gb. The sequencing was carried out on the Illumina NovaSeq. 6000 platform at Novogene. Detailed information on the metagenomic and meta‐transcriptomic datasets is listed in Table S1.

### MAG recovery

MAGs were recovered from NC metagenomes through a strategy established in our previous study [58, 59]. As outlined in Figure 1, the raw Illumina reads were filtered by fastp (v0.23.2) [60]. The clean reads were assembled into contigs using metaSPAdes (v3.15.5) [61]. MAGs were retrieved from the assembled contigs via a binning approach using BASALT (v1.0.0) [62]. The completeness and contamination of MAGs were evaluated by CheckM (v1.2.2) [63]. MAGs were dereplicated using dRep (v3.4.2) [64] with the following parameters: “‐sa 0.95 ‐comp 40 ‐con 20 ‐nc 0.30.” The MAGs were classified using GTDB‐Tk (v2.1.1) [65]. The relative abundance of MAGs in each sample was expressed as reads per kilobase per million mapped reads (RPKM) values, which were calculated by CoverM (v0.6.1) (https://github.com/wwood/CoverM) with the following parameters: “genome ‐‐min‐read‐aligned‐percent 75 ‐‐min‐read‐percent‐identity 95 ‐‐min‐covered‐fraction 75 ‐m rpkm.”

### Viral contig identification and quantification

Viral contigs were identified from metagenomes via the pipeline shown in Figure 1. Only linear contigs ≥5 kb or circular contigs ≥1.5 kb were piped into viral identification. Candidate viral contigs were identified from assembled contigs by four tools, including DeepVirfinder (v1.0) [66], Virsorter2 (v2.2.4) [67], VIBRANT (v1.2.1) [68], and viralVerify (v1.1) [69] in parallel. Contigs with a max score ≥ 0.8 or a score ≥ 0.8 and *p* < 0.05 were recognized as candidate viral contigs by VirSorter2 or DeepVirfinder, respectively. The combined set of candidate viral contigs from these tools underwent further analysis. The quality and completeness of candidate viral contigs were assessed by CheckV (v1.0.1) [70]. The viral proteins in candidate viral contigs were identified by CheckV and CAT (v5.2.3) [71]. Only candidate viral contigs containing more than one viral protein were retained. These filtered viral contigs were dereplicated and clustered into viral operational taxonomic units (vOTUs) using MMseqs. 2 (v14‐7e284) [72] with the following parameters: “‐‐min‐seq‐id 0.95 ‐c 0.8 ‐‐cov‐mode 1 ‐e 1E‐05 ‐‐cluster‐mode 2 ‐‐cluster‐reassign.” The relative abundance of vOTUs in each sample was expressed as RPKM values, which were calculated by CoverM (v0.6.1) with the following parameters: “contig ‐‐min‐read‐aligned‐percent 75 ‐‐min‐read‐percent‐identity 95 ‐‐min‐covered‐fraction 75 ‐‐contig‐end‐exclusion 0 ‐m rpkm.” Shannon and Pielou's evenness indices for the microbial community were computed using R Project (v4.3.0). Principal coordinates analysis (PCoA) based on Bray–Curtis distances was conducted to evaluate community dissimilarities.

### Viral contig classification and host analysis

The taxonomic classification of viral contigs was performed by three methods based on the International Committee on Taxonomy of Viruses (ICTV) taxonomy rules. First, PhaGCN2 (v2.1) [73] was applied for taxonomic classification. The remaining unclassified contigs were annotated by CAT (v5.2.3) and then subjected to BLASTn against the IMG/VR database (v4) [74]. The lifestyle of each bacterial virus was predicted by PhaTYP [75]. Viral hosts were categorized based on their taxonomy. The infection associations between prokaryotic viruses and prokaryotes were further predicted based on CRISPR spacer match and shared genomic contents. CRISPR spacers from each MAG were extracted using CRISPRCasTyper (v1.8.0) [76]. These CRISPR spacers were then matched against viral contigs by BLASTn with the following settings: “‐task blastn‐short ‐perc_identity 100 ‐penalty ‐1 ‐gapopen 10 ‐gapextend 2 ‐word_size 7.” Direct matching of viral contigs to MAGs was also performed to pinpoint their hosts using BLASTn with parameters: “‐perc_identity 70 ‐qcov_hsp_perc 75 ‐evalue 1e‐3.”

### Identification of AMGs and ARGs

The open reading frames (ORFs) of contigs were predicted by Prodigal (v2.6.3) [77]. High‐quality viral contigs, as determined by CheckV (v1.0.1) [70], were analyzed for AMGs using VIBRANT (v1.2.1) [68]. ARGs were identified via AMRfinderPlus (v3.11.2) [78] and BLASTp against the SARG database (v3.1) [79] with a similarity cutoff of 80% and alignment length cutoff of 70%. The abundance of these genes was quantified by CoverM (v0.6.1) with the following parameters: “contig ‐‐min‐read‐aligned‐percent 75 ‐‐min‐read‐percent‐identity 95 ‐‐min‐covered‐fraction 75 ‐‐contig‐end‐exclusion 0 ‐m rpkm.”

### Meta‐transcriptomic analysis of viruses

Raw meta‐transcriptomic reads were filtered by fastp (v0.23.2) [60]. Clean meta‐transcriptomic reads were mapped to viral contigs or selected genes by Bowtie2 (v2.5.1) [80]. The mapped reads on each viral contig were counted by SAMtools (v1.16.1) [81]. Expression levels of selected genes were calculated by RSEM (v1.3.3) [82] and normalized to transcripts per million (TPM).

### Statistical analysis and visualization

Statistical analyses and visualization were conducted using the R Project (v4.3.0). To assess significant differences between groups, a one‐way analysis of variance (ANOVA) or Student's *t* test was conducted. For post hoc analyses in ANOVA, Tukey's test was applied to perform multiple comparisons.

## AUTHOR CONTRIBUTIONS

Bing Li and Jiayu Zhang conceived the project and designed the experiments. Jiayu Zhang did the experiments, analyzed data, and wrote the manuscript. Bing Li supervised the project and revised the manuscript. Aixi Tang contributed to the methodology. Tao Jin and Wensheng Shu contributed to the sequencing of viromes. Deshou Sun, Fangliang Guo, and Huaxin Lei contributed to sample collection and nucleic acid extraction. Pingfeng Yu contributed to the methodology and reviewed the manuscript. Xiaoyan Li and Lin Lin supervised the project. All authors have read the final manuscript and approved it for publication.

## CONFLICT OF INTEREST STATEMENT

The authors declare no conflict of interest.

## ETHICS STATEMENT

No animals or humans were involved in this study.

## Supporting information


**Figure S1:** Viral contig number and read mapping rate to viral contigs in each WWTP unit revealed by VPC and NC metagenomics.
**Figure S2:** Taxonomic classification of viral contigs identified in VPC and NC metagenomes.
**Figure S3:** Dynamics of different nucleic acid types of viruses across the wastewater treatment processes.
**Figure S4**: Distribution of viruses with extremely small and large sizes in the NC and VPC samples.
**Figure S5**: Dynamics of phages with different lifestyles across the wastewater treatment processes.
**Figure S6**: Principal coordinates analysis (PCoA) of viral diversity in three WWTPs.
**Figure S7**: Transcriptional expression of eight representative viral contigs.
**Figure S8**: AMGs harbored by high‐quality viral contigs.
**Figure S9**: Arrangement of representative ARGs in viral contigs.

**Table S1:** Information of samples and sequencing datasets from the three WWTPs.

## Data Availability

(All the sequencing data have been deposited in NCBI under BioProject accession number PRJNA1065278 http://www.ncbi.nlm.nih.gov/bioproject/PRJNA1065278/). The data and scripts used are saved in GitHub http://github.com/Z-bioinfo/2024iMeta. Supplementary materials (figures, tables, scripts, graphical abstract, slides, videos, Chinese translated version, and update materials) may be found in the online DOI or iMeta Science http://www.imeta.science/.
