# Supplementary material for: A panoramic view of the virosphere in three wastewater treatment plants by integrating viral‐like particle‐concentrated and traditional non‐concentrated metagenomic approaches
Source: Imeta. 2024 Mar 29;3(3):e188. doi: 10.1002/imt2.188 (PMC11183165; doi:10.1002/imt2.188)
Supplement: Supplementary file 1 — Figure S1: Viral contig number and read mapping rate to viral contigs in each WWTP unit revealed by VPC and NC metagenomics. Figure S2: Taxonomic classification of viral contigs identified in VPC and NC metagenomes. Figure S3: Dynamics of different nucleic acid types of viruses across the wastewater treatment processes. Figure S4: Distribution of viruses with extremely small and large sizes in the NC and VPC samples. Figure S5: Dynamics of phages with different lifestyles across the wastewater treatment processes. Figure S6: Principal coordinates analysis (PCoA) of viral diversity in three WWTPs. Figure S7: Transcriptional expression of eight representative viral contigs. Figure S8: AMGs harbored by high‐quality viral contigs. Figure S9: Arrangement of representative ARGs in viral contigs. [file IMT2-3-e188-s002.pdf]

Supplementary information to

A panoramic view of the virosphere in three wastewater treatment plants treating different types of wastewater by integrating viral-like particle-concentrated and traditional non-concentrated metagenomic approaches

Running title: Comparison of two methods for WWTP virome

Jiayu Zhang[1,2], Aixi Tang[1], Tao Jin[3], Deshou Sun[1,5], Fangliang Guo[1], Huaxin Lei[1], Lin Lin[1], Wensheng Shu[3,4], Pingfeng Yu[6], Xiaoyan Li[1], Bing Li[1*]

[1] Institute of Environment and Ecology, Tsinghua Shenzhen International Graduate School, Tsinghua University, Shenzhen, 518055, China.

[2] Research Center for Eco-environmental Engineering, Dongguan University of Technology, Dongguan, 523808, China

[3] Guangdong Magigene Biotechnology Co., Ltd., Shenzhen, 518055, China

[4] Institute of Ecological Science, Guangzhou Key Laboratory of Subtropical Biodiversity and Biomonitoring, Guangdong Provincial Key Laboratory of Biotechnology for Plant Development, School of Life Sciences, South China Normal University, Guangzhou, 510631, China

[5] Shenzhen Tongchen Biotechnology Co., Limited, Shenzhen, 457001, China.

[6] College of Environmental and Resource Sciences, Zhejiang University, Hangzhou, 310058, China

*Correspondence: bingli@sz.tsinghua.edu.cn (Bing Li)

This file includes:

Supplemental Figures:

Figure S1 Viral contig number and read mapping rate to viral contigs in each WWTP unit revealed by VPC and NC metagenomics.

Figure S2 Taxonomic classification of viral contigs identified in VPC and NC metagenomes.

Figure S3 Dynamics of different nucleic acid types of viruses across the wastewater treatment processes.

Figure S4 Distribution of viruses with extremely small and large size in the NC and VPC samples.

Figure S5 Dynamics of phages with different lifestyles across the wastewater treatment processes.

Figure S6 Principal coordinates analysis (PCoA) of viral diversity in three WWTPs.

Figure S7 Transcriptional expression of eight representative viral contigs.

Figure S8 AMGs harbored by high-quality viral contigs.

Figure S9 Arrangement of representative ARGs in viral contigs.

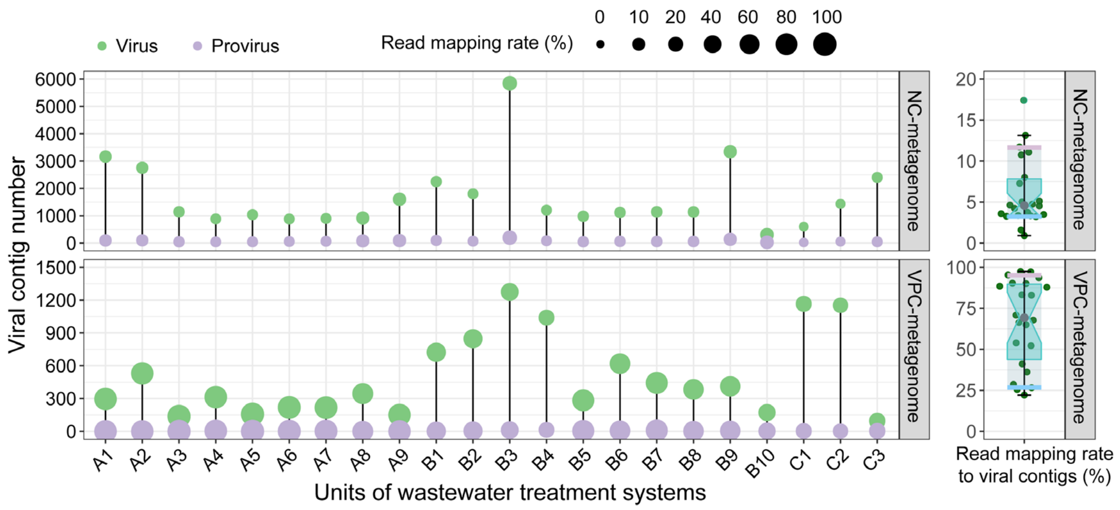

Figure S1 Viral contig number and read mapping rate to viral contigs in each WWTP unit revealed by VPC and NC metagenomics.

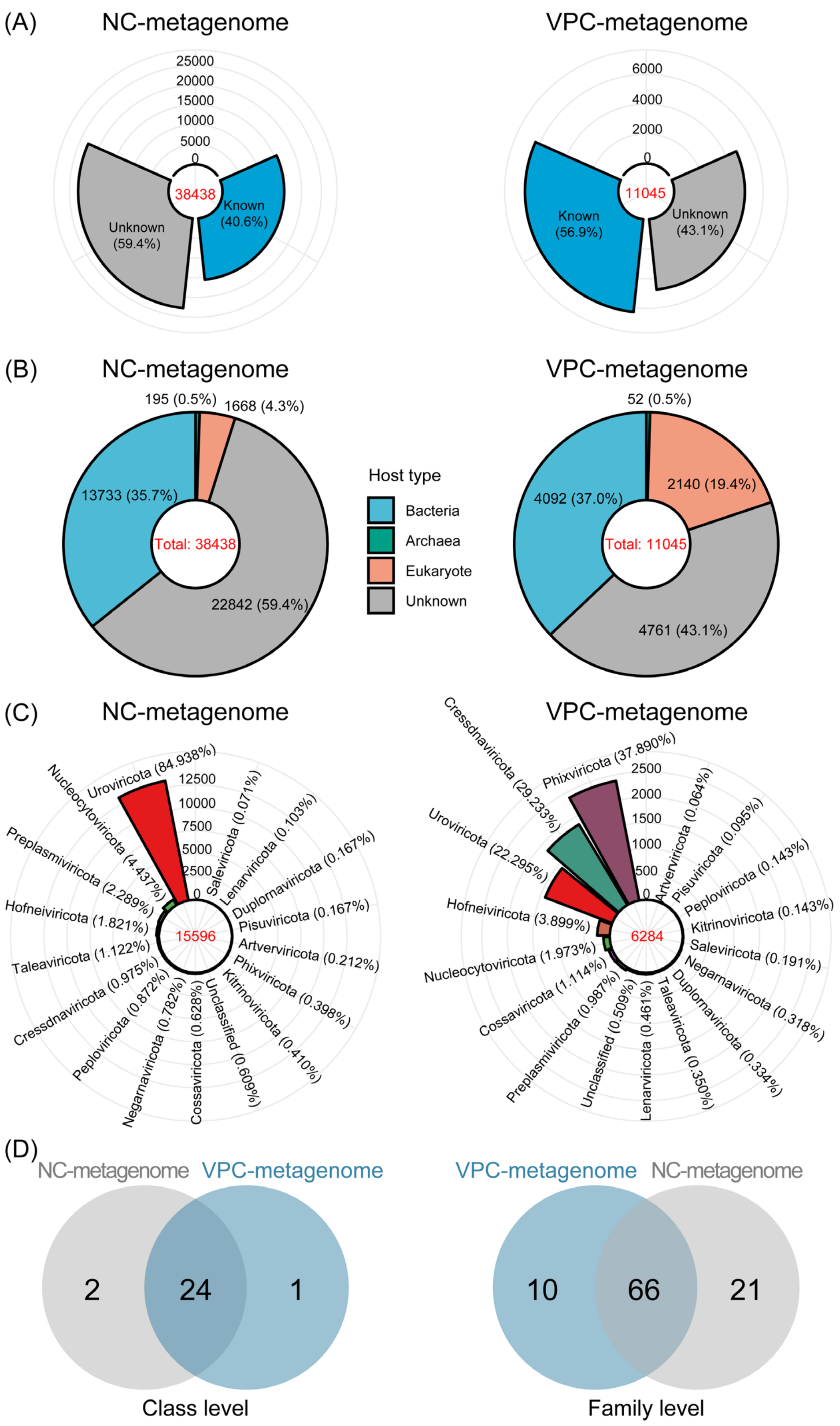

Figure S2 Taxonomic classification of viral contigs identified in VPC and NC metagenomes. (A) Proportion of classified viral contigs from VPC and NC metagenomes. **(B)** Host types of viral contigs. **(C)** Taxonomic composition at the phylum level. **(D)** Intersection of taxonomies at class and family levels.

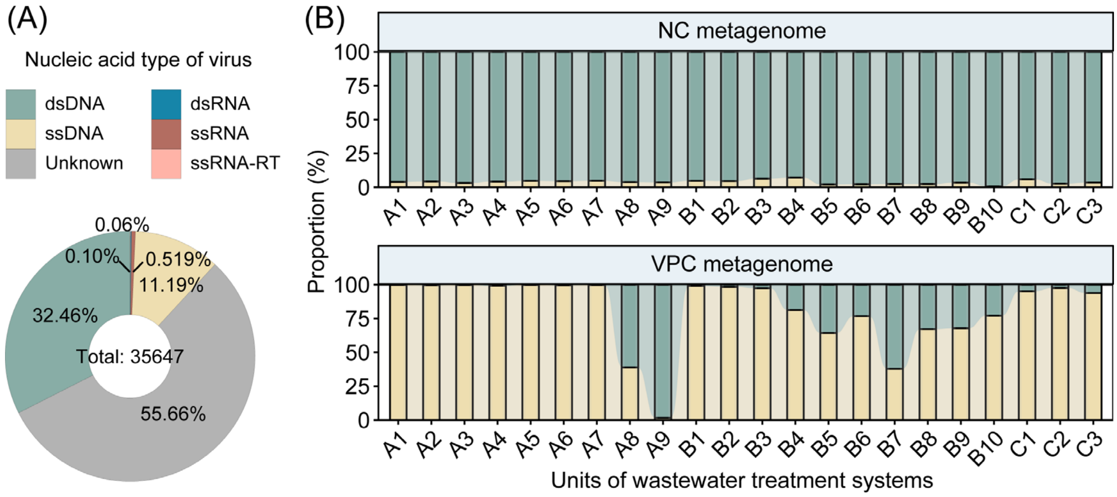

Figure S3 Dynamics of different nucleic acid types of viruses across the wastewater treatment processes. (A) vOTU number of each nucleic acid type of virus. **(B)** Proportion of dsDNA and ssDNA viruses. Proportion was calculated as the percentage of dsDNA or ssDNA virus abundance in the sum of both.

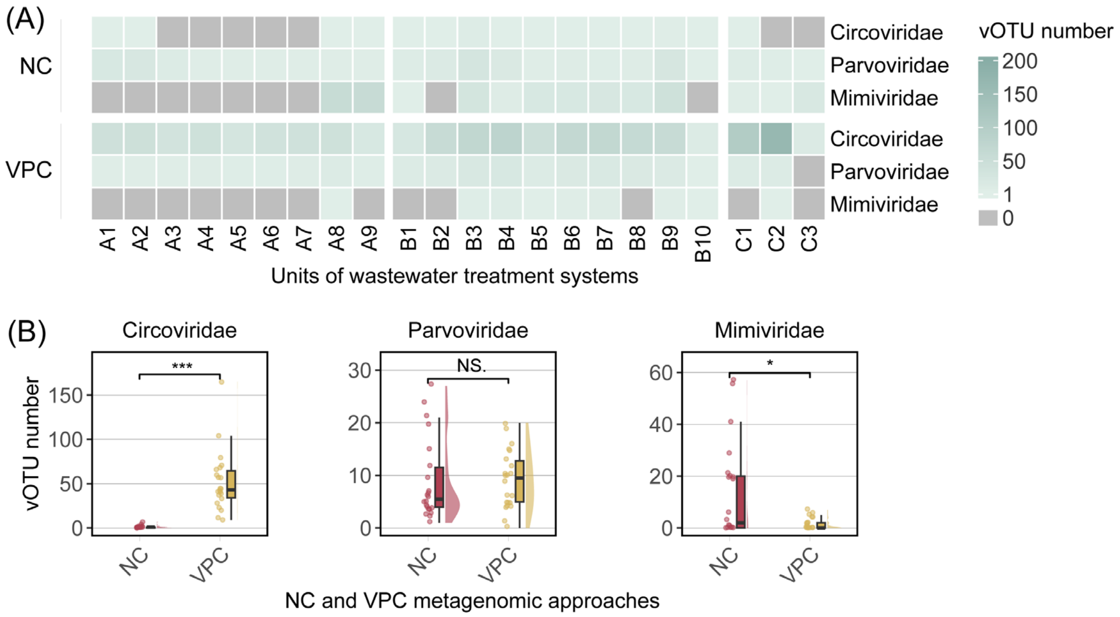

Figure S4 Distribution of viruses with extremely small and large size in the NC and VPC samples. (A) vOTU number of typical viruses with extremely small and large size in the NC and VPC samples. **(B)** Comparison of vOTU number of typical viruses with extremely small and large sizes between NC and VPC metagenomes. NS. indicates no significant difference.

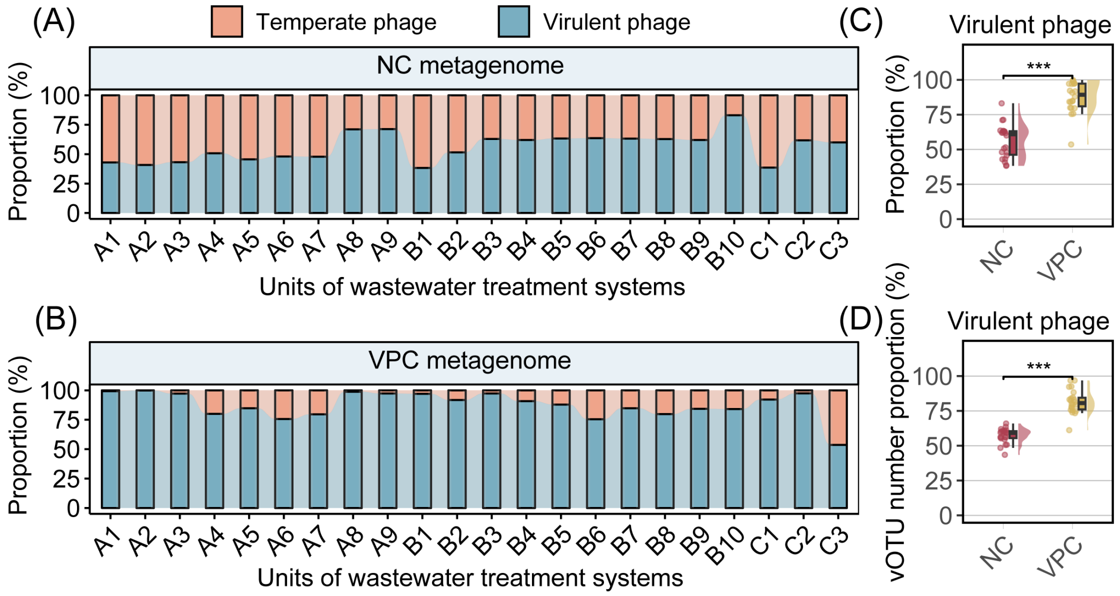

Figure S5 Dynamics of phages with different lifestyles across the wastewater treatment processes. (A, B) Proportion of virulent and temperate phages in NC **(A)** and VPC **(B)** metagenomes. Proportion was calculated as the percentage of virulent or temperate phage abundance in the sum of both. **(C)** Comparison of proportion of virulent phage abundance between NC and VPC metagenomes. **(D)** Comparison of proportion of virulent vOTU number between NC and VPC metagenomes.

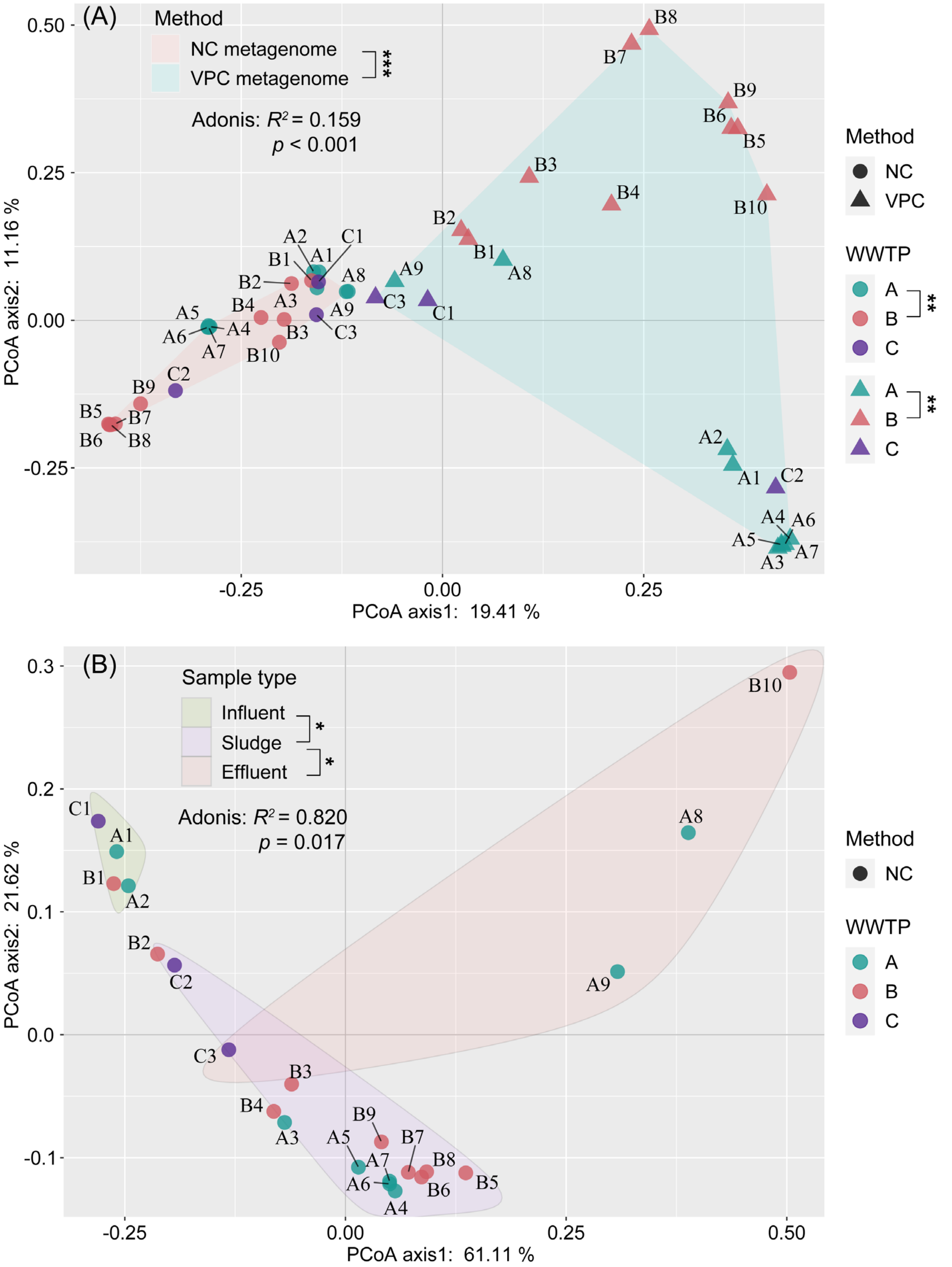

Figure S6 Principal coordinates analysis (PCoA) of viral diversity in three WWTPs. (A) PCoA analysis of viral diversity at the vOTU level based on Bray-Curtis distance. The significance of difference in beta diversity of VPC and NC samples was determined by adonis analysis. **(B)** PCoA analysis of viral diversity at the family level in NC metagenomes. Adonis analysis was employed to determine the significance of difference in beta diversity of influent (A1, B1, and C1), sludge (A4, A5, A6, A7, B5, B6, B7, B8, and C2), and effluent samples (A9, B10, and C3) across the three WWTPs (A: duckery, B: swine, C: municipal wastewater).

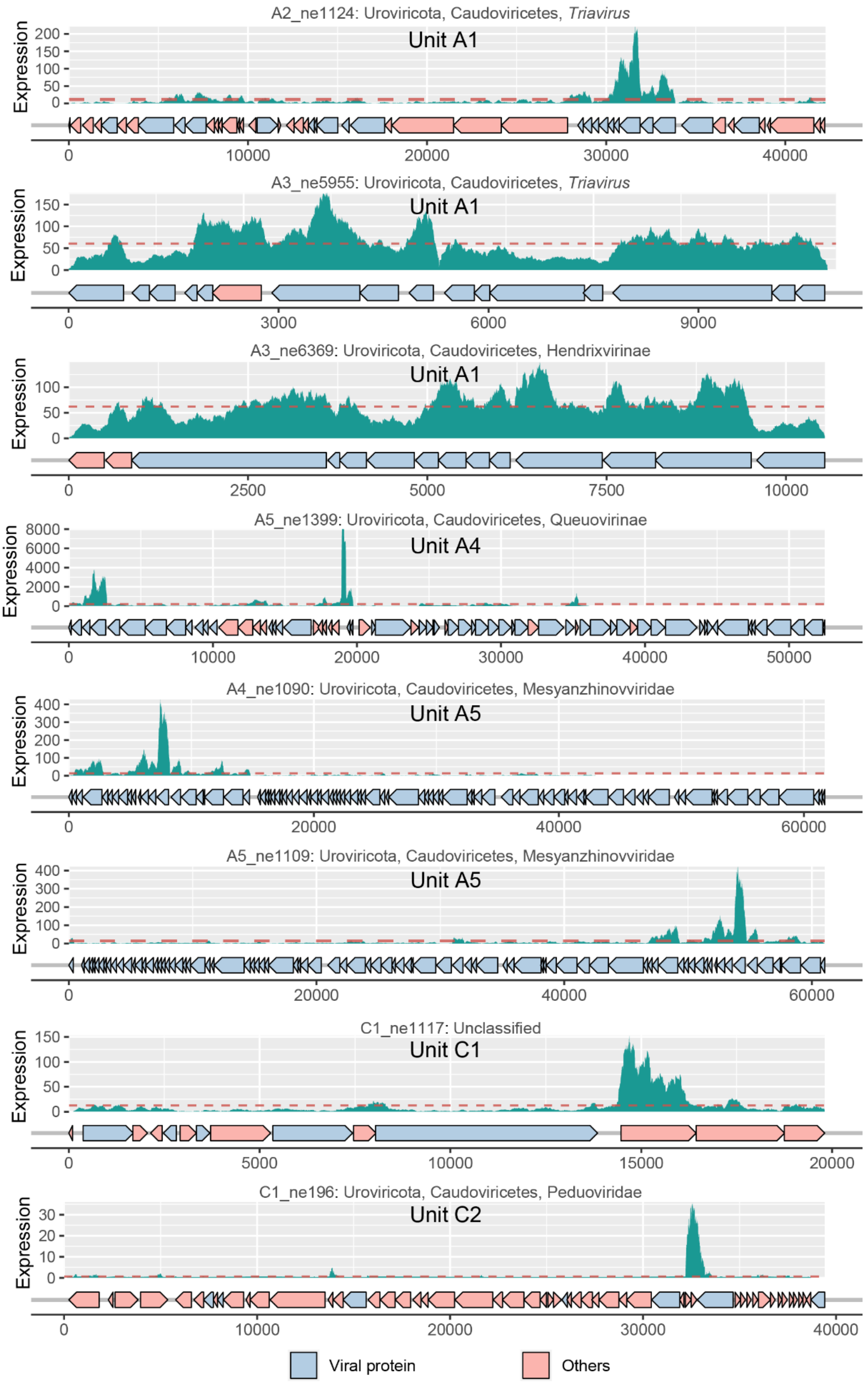

Figure S7 Transcriptional expression of eight representative viral contigs. Values are presented as mean ± SD (n=3) of transcriptional expression (mapped reads per base). The red dashed line indicates the average transcriptional expression of a viral contig.

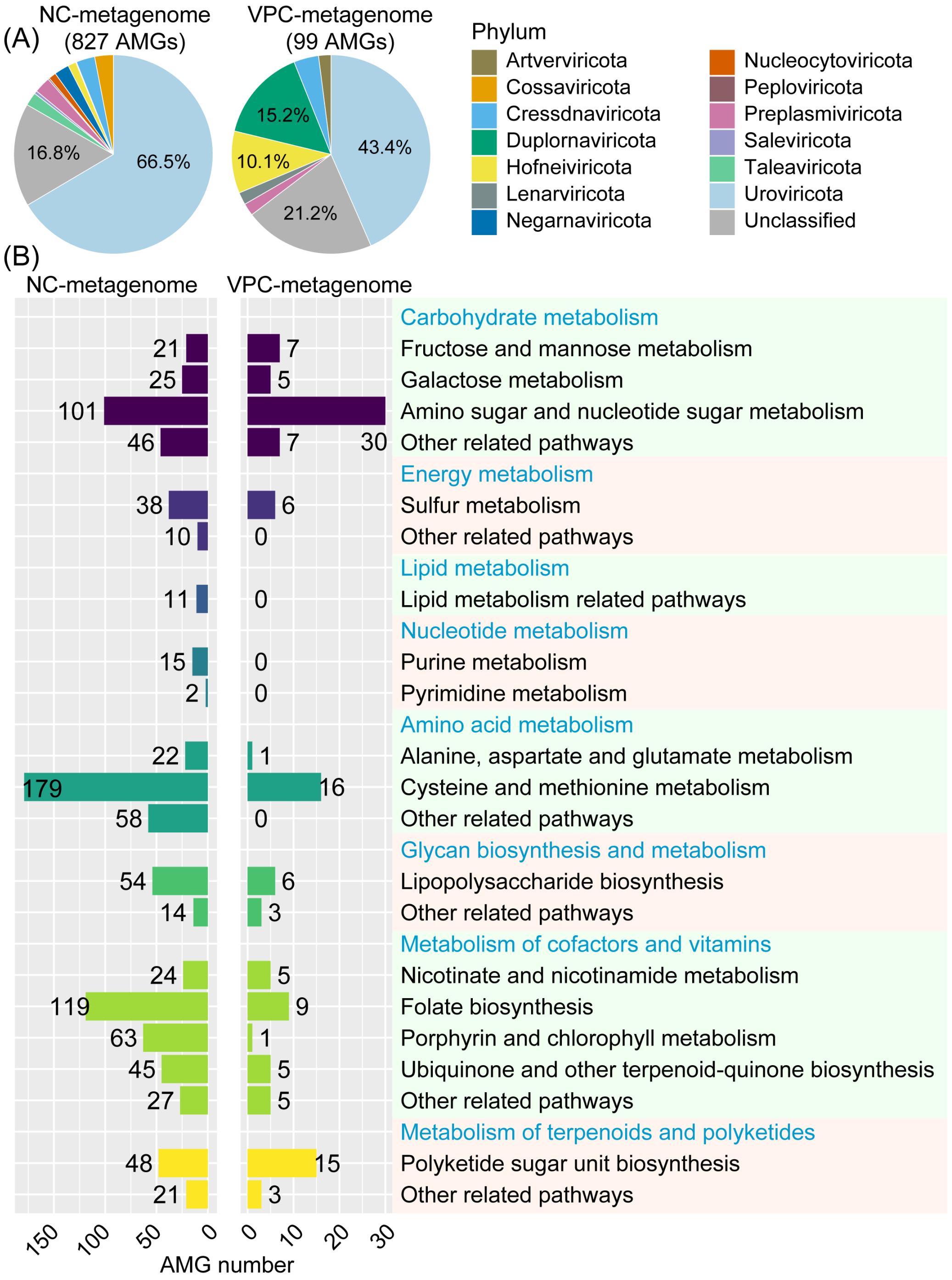

Figure S8 AMGs harbored by high-quality viral contigs. (A) Taxonomic composition of viral contigs harboring AMGs. **(B)** Metabolic pathways involving these AMGs.

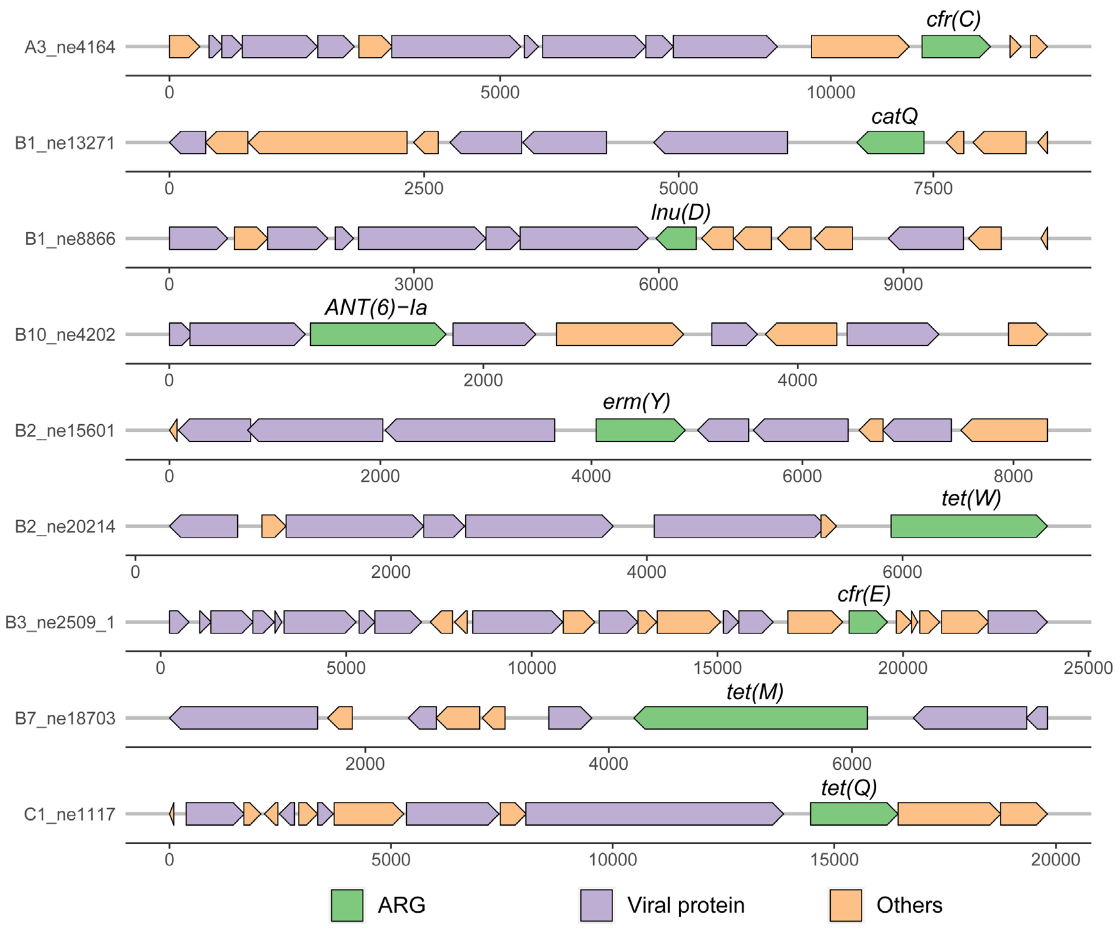

Figure S9 Arrangement of representative ARGs in viral contigs.
